# Comparison of different cementing techniques for cement penetration under tibial component in total knee arthroplasty: a retrospective observational study

**DOI:** 10.1186/s43019-024-00232-7

**Published:** 2024-09-20

**Authors:** Yu Okuno, Keita Nagira, Koji Ishida, Haruhisa Kanaya, Ikuta Hayashi, Makoto Enokida, Hideki Nagashima

**Affiliations:** Department of Orthopedic Surgery, Faculty of Medicine, Tottori University, 36-1 Nishi-Cho, Yonago, Tottori 683-8504 Japan

**Keywords:** Knee, Osteoarthritis, Total knee arthroplasty, Cementing technique, Hounsfield unit value

## Abstract

**Purpose:**

This study aimed to investigate the differences in cement penetration between cementing techniques in total knee arthroplasty (TKA).

**Materials and methods:**

We retrospectively evaluated knee undergone TKA at our hospital for both preoperative and postoperative computed tomographic (CT) evaluations. Cementing was performed with hand mixing and hand packing (HM group) and with vacuum mixing and cement gun use (VM group). We measured the area under the tibial baseplate (sclerotic and nonsclerotic sides) and compared the mean and maximum depths of cement penetration at each area.

**Results:**

Of the 44 knees evaluated, 20 and 24 knees were in the HM and VM groups, respectively. At the center of the sclerotic side, the mean penetration depths (2.0 ± 0.7 and 2.5 ± 0.7 mm, *p* = 0.02) and the maximum penetration depths (4.0 ± 0.9 and 5.0 ± 1.6 mm, *p* = 0.02) were significantly deeper in the VM group than in the HM group. The correlation between preoperative Hounsfield unit values and mean penetration were *r* = –0.617 (*p* < 0.01) and –0.373 (*p* = 0.01) in the HM and VM groups, respectively.

**Conclusion:**

The cementing technique of vacuum mixing and using a cement gun allowed for deeper cement penetration compared with the hand mixing and hand packing technique, even in bone sclerotic sites.

## Introduction

Total knee arthroplasty (TKA) survival rate within 10 years after surgery has improved [1]; however, the number of TKA revisions in France increased approximately 6.5-fold between the periods 1991–1998 and 2013–2016 [2]. In the USA, the number of revision procedures was expected to increase by 601% between 2005 and 2030 [3]. Therefore, a concern is how to prevent the need for revision TKA. Infection is the primary reason for revision in TKA (25.2%), and the second most common reason is reported to be aseptic loosening (24.1%) [4].

In TKA, cement fixation initially performs better and results in less settlement of the tibial baseplate compared with cementless fixation [5]. Insufficient cement penetration of the bone increases the risk of aseptic loosening. Thus, we believe that ensuring adequate cement penetration below the tibial baseplate is crucial for reducing the need for revision. However, despite the introduction of various cementing techniques in TKA, a gold standard method has yet to be established.

In most previous reports, the evaluations of cement penetration were based on radiographs. Only one study was based on computed tomography (CT) measurements: Verburg et al. [6] investigated cement penetration under the tibial baseplate by using CT scans obtained after TKA. However, their method involved measuring the area of penetration on a postoperative horizontal CT slice. Furthermore, we found no literature on measuring the maximum depths of penetration on CT images, and correlations among cementing technique, cement penetration, and preoperative Hounsfield unit (HU) values have not been reported. The HU is a relative quantitative measurement of CT images, calculated through a linear transformation of the baseline linear attenuation coefficient of the X-ray beam. Distilled water (at standard temperature and pressure) is arbitrarily defined as zero HU, whereas air is defined as –1000 HU. Denser tissue, with greater X-ray beam absorption, yields positive values and appears bright, whereas less dense tissue, with lower X-ray beam absorption, results in negative values and appears dark [7, 8]. Recently, studies have reported a correlation between bone mineral density and the HU values [9, 10].

In this study, we used postoperative CT scans to investigate the penetration of cement into the bone below the tibial baseplate in TKA with different cementing techniques. We also evaluated whether preoperative HU values at the cement injection site were associated with cement penetration.

## Materials and methods

A total of 48 patients underwent TKA (in 58 knees) at our hospital from July 2017 to December 2019, and we retrospectively studied those who underwent both preoperative and postoperative CT evaluation. Patients underwent the procedure with one of two cementing techniques: hand mixing and hand packing (the HM group), between July 2017 and December 2018, or vacuum mixing and use of a cement gun (the VM group), between January and December 2019. The medial parapatellar approach or midvastus approach was used as the surgical technique. A tourniquet was used in all cases, and before bone cementation, the bone was cleaned with pulse lavage and then dried, and fat and blood were removed with CarboJet^®^ (Kinamed, Camarillo, CA, USA) [11]. Anchor holes were created using a 3.2-mm drill bit (Zimmer Biomet, Warsaw, IN, USA) in the sclerotic subchondral bone [12]. We used Persona^®^ implants (Zimmer Biomet, Warsaw, IN, USA) in all cases; the bone cement was Cobalt HV Bone Cement or Cobalt G-HV Bone Cement (with gentamicin; both by Zimmer Biomet). The cement was applied to the implant, the surface of tibia and femur in that order. In the HM group, cement was applied after the mixing for 30 s to 1 min and after the cement was no longer sticky. In the VM group, the cement was applied immediately after the mixing. The cement gun was an Optivac M (Zimmer Biomet), and a 23-Degree Pressurizing Nozzle (Zimmer Biomet) was attached to the tip of the cement gun for application. The tip of this nozzle was cut at an angle, and by pressing the tip of the nozzle against the bone surface, the cement injection surface became perpendicular to the bone, which is considered advantageous for cement penetration into the bone.

**Fig. 1:**
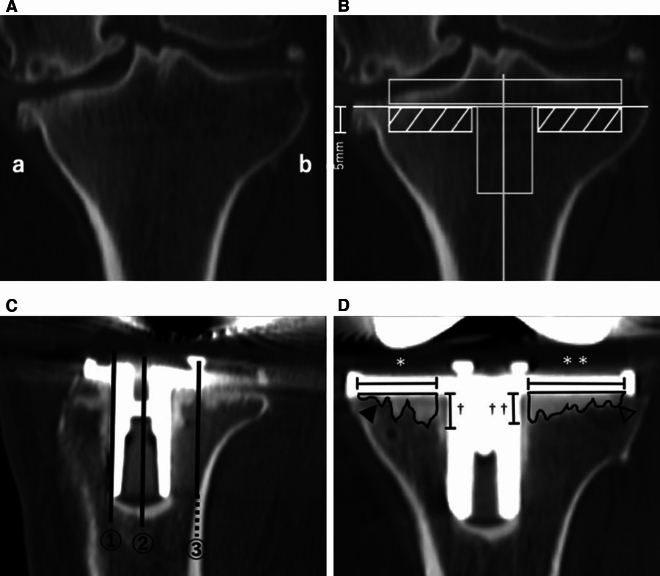
Evaluation method of pre- and postoperative Hounsfield unit (HU) values and cement penetration in computed tomography (CT) images. **A** Preoperative CT coronal section. (**a**) Sclerotic side and (**b**) nonsclerotic side. **B** Preoperative CT coronal section. Vertical and horizontal lines represent the tibial axis and the osteotomy line in operation, respectively. The shaded area is the estimated area of bone penetration by cement and was defined as the length of the base plate multiplied by 5 mm (the average HU values in the area are calculated by the imaging software). **C** Postoperative CT sagittal section. (1) Anterior edge of the keel, (2) center of the keel, and (3) extension of the posterior cortex. **D** Postoperative CT coronal section. The solid arrowhead indicates the cement penetration area of the sclerotic side (mm^2^), The open arrowhead indicates the cement penetration area of the nonsclerotic side (mm^2^). The single asterisk indicates the base of the sclerotic side (mm). The double asterisk indicates the base of the nonsclerotic side (mm). The single dagger presents the maximum penetration in the sclerotic side (mm). The double dagger refers to the maximum penetration in the nonsclerotic side (mm). Mean penetration: area/base (mm)

**Fig. 2:**
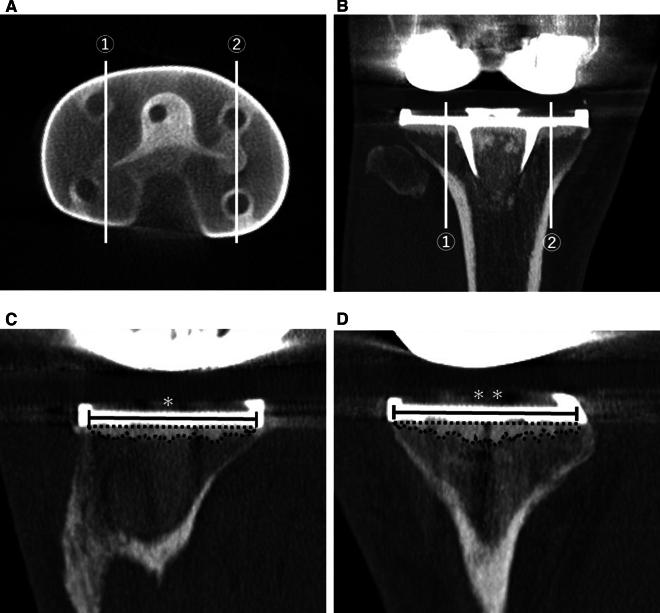
Evaluation method of mean cement penetration on sagittal section in postoperative computed tomography (CT) image. **A** Postoperative CT axial section just under the tibial baseplate. **B** Postoperative CT coronal section at the most posterior level of the fin. In (**A**) and (**B**), the (1) lateral side of sagittal section and (2) the medial side of sagittal section level are shown. The level of the sagittal section was one slice lateral to the section in which the most posterior portion of the fin of the tibial component was delineated for the medial and lateral sides. **C** Postoperative CT sagittal section of the lateral side. **D** Postoperative CT sagittal section of the medial side. In (**C**) and (**D**), the area surrounded by the black dotted line represents the cement penetration area (mm^2^). The single and double asterisks indicates the base of the lateral side (mm) and the base of the medial side (mm), respectively. Mean penetration: area/base (mm)

**Fig. 3:**
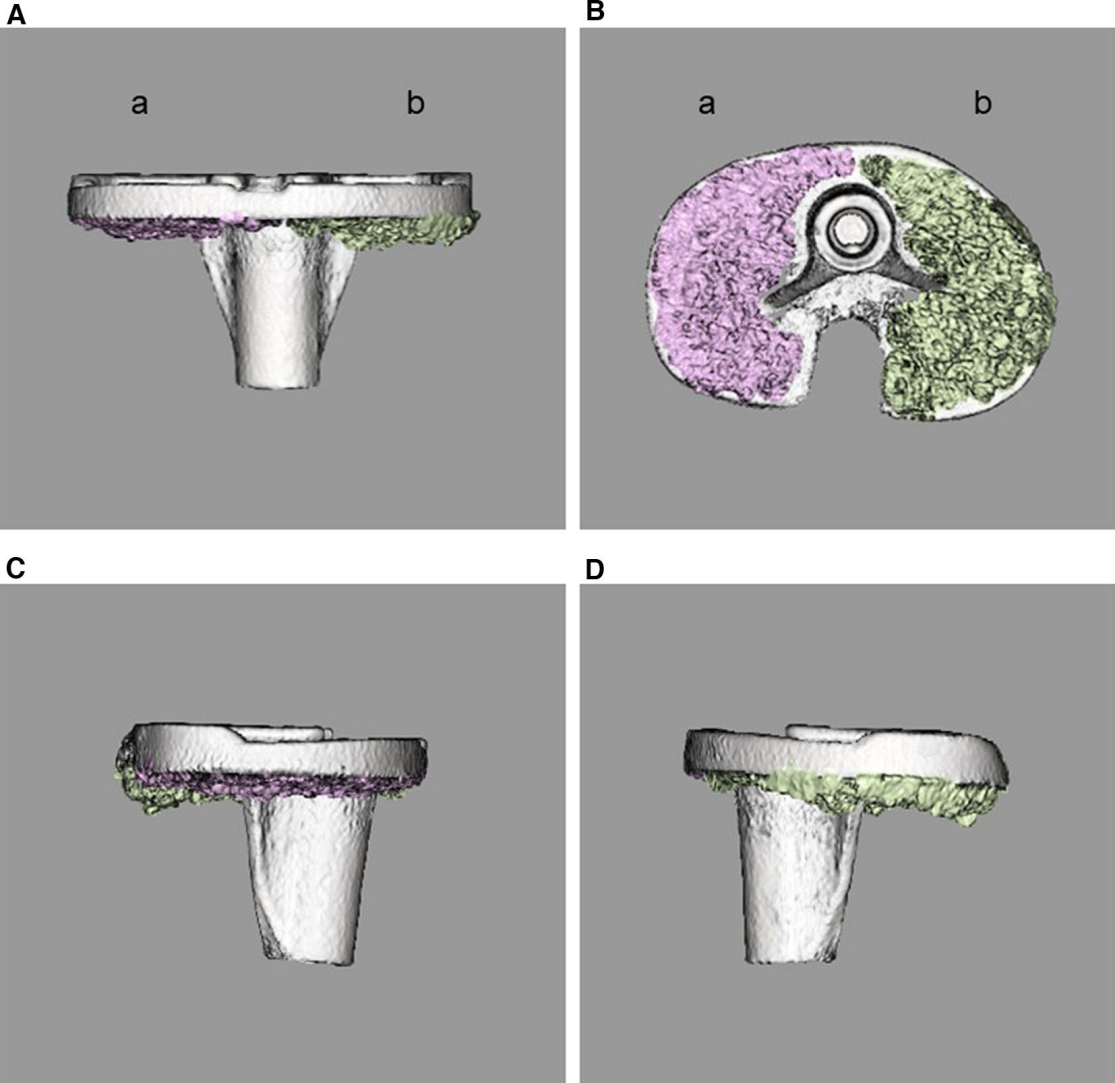
Three-dimensional computed tomography (3D CT) image of the cement penetration area. **A** Postoperative 3D CT frontal view. **B** Postoperative 3D CT caudal view. In (**A**) and (**B**), (**a**) lateral side and (**b**) the medial side are shown. **C** Postoperative 3D CT lateral view from lateral side. **D** Postoperative 3D CT lateral view from medial side. The maximum penetration in 3D CT was defined as the distance from the baseplate to the most penetrated area in each true lateral view. In any images, cement surrounding 2 mm around the keel and fin is excluded when creating the 3D CT image to prevent overestimation of cement penetration

We documented patient characteristics such as age, sex, preoperative diagnosis, Kellgren–Lawrence (K–L) grade, and preoperative femorotibial angle (FTA), and we recorded the type of cement used. We also calculated the mean and maximum depths of cement penetration under the tibial baseplate and preoperative HU values of bone from preoperative and postoperative CT scans. To examine the mean depths of cement penetration, the side with more arthropathic changes was defined as the sclerotic side on preoperative CT and the contralateral side as the normal side (Fig. 1A). We calculated the mean HU values of a rectangular area of 5 mm × the length of the baseplate by matching the preoperative and postoperative CT scans according to the baseplate placement angle and the amount of osteotomy during surgery (Fig. 1B). The anterior part was defined as the anterior edge of the keel of the tibial baseplate, the central part as the center of the keel, and the posterior part as the extension of the posterior tibial cortex (Fig. 1C). The mean and maximum depths of penetration were measured in the postoperative CT coronal sections for the anterior, central, and posterior parts of the sclerotic and nonsclerotic sides. We used bone morphometric measurements, whereby mean penetration (in millimeters) was the cement area below the tibial baseplate (in square millimeters) divided by the base (in millimeters) [13, 14]. We defined “maximum penetration” (in millimeters) as the deepest penetration into the cancellous bone from the tibial baseplate, in accordance with previous evaluations of radiographs [15] (Fig. 1D). To avoid measuring bone cement applied to the keel before implant insertion, we excluded 2 mm on both sides of the keel from the measurement area. Similarly, the mean penetration was evaluated in the sagittal section. The level of the sagittal section was one slice lateral to the section in which the most posterior portion of the fin of the tibial component was delineated for the medial and lateral sides (Fig. 2). In addition, 3D CT was used to evaluate the maximum penetration of each of the sclerotic and nonsclerotic sides. The 3D CT images were created with postoperative CT when removing the cement that penetrated the anchor hole and the 2-mm cemented area around the keel and fin and were evaluated from the true lateral side of the sclerotic and nonsclerotic sides. The distance from the baseplate to the most penetrated area was defined as the maximum penetration (Fig. 3). Penetration was calculated from the area measured on the medical imaging information system SYNAPSE (Fujifilm Holdings Corporation, Tokyo, Japan); the CT window width was set to 3000 HU and the window level to 1000 HU to clarify the bone–cement interface. The areas were measured independently by two examiners under identical conditions.

**Fig. 4:**
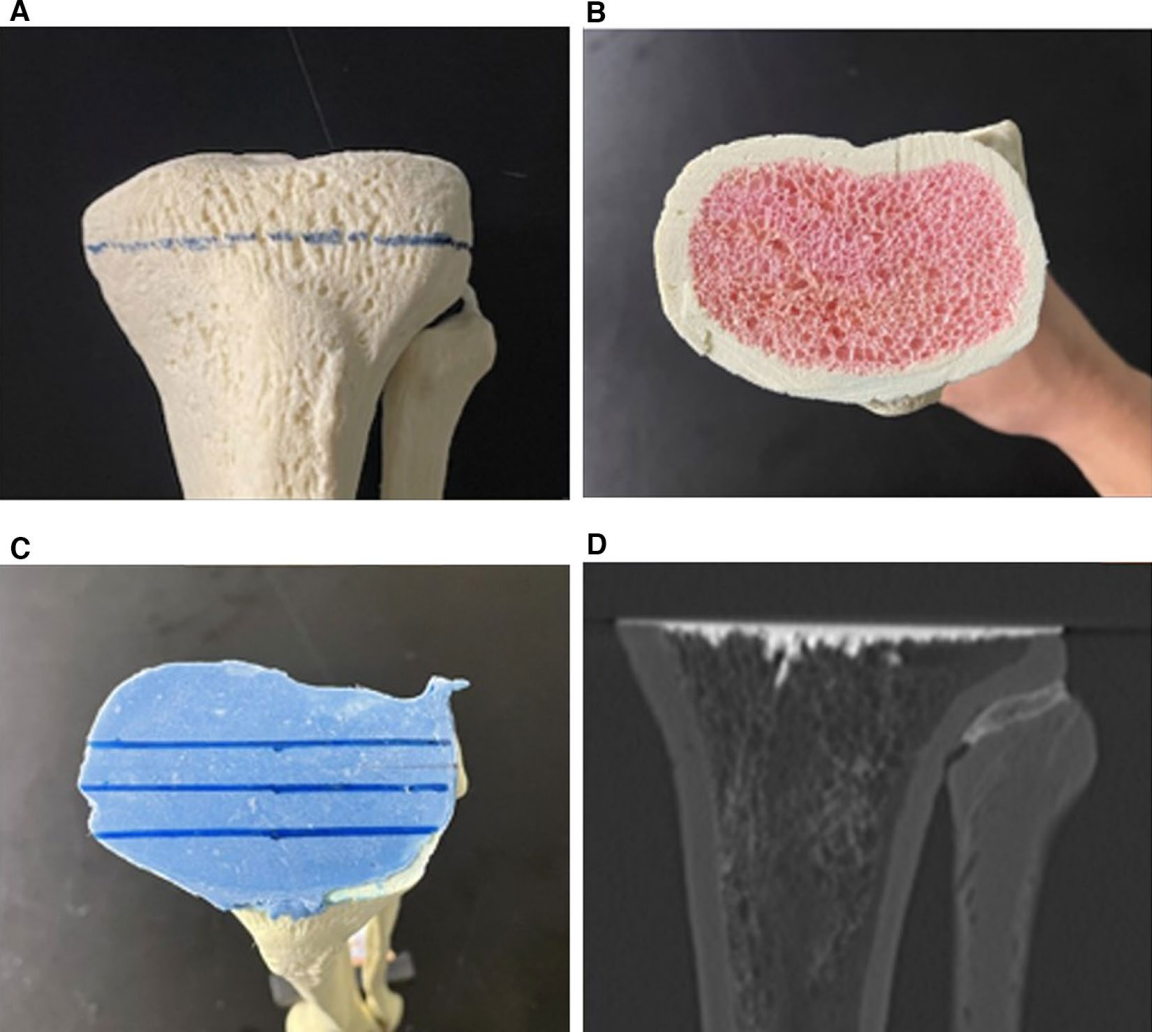
Preliminary study to evaluate cement penetration using a bone model. **A** Bone model of tibia. The osteotomy line was set as observed in total knee arthroplasty. **B** Osteotomy surface of the bone model. **C** Bone model after cement application. Cement penetration was measured at each of the cross-sections where the lines were drawn. **D** Computed tomographic assessment on the bone model. The same method as in Fig. 1 was used to evaluate the mean and maximum cement penetration

In addition, we conducted a preliminary study using a bone model to evaluate the effect of antibiotic content on cement penetration with CT scans (Fig. 4). As in the case of TKA, cement was applied to the osteotomy surface of the bone model, and the cement was pressed into the bone model using a metal plate as a baseplate. We evaluated the cement penetration using CT and set the imaging conditions, CT window width, and window level the same as those in the present study.

### Statistical analysis

XLSTAT (Addinsoft, Paris, France) and SPSS (IBM Corporation, Armonk, NY, USA) were used for statistical analyses. The *χ*^2^ test was used to analyze sex, preoperative diagnosis, K–L grade, and the type of cement; Student’s *t*-test was used to analyze age, preoperative FTA, and mean and maximum depths of penetration; and Pearson’s correlation coefficient test was used to analyze the correlation between preoperative HU values and mean depths of penetration. The significance level was set at 0.05. In the intraclass correlation coefficient (ICC) test for area measurement, intrarater reliability showed ICC (1, 2) = 0.768 with Cronbach’s *α* = 0.869, whereas interrater reliability demonstrated ICC (2, 2) = 0.687 with Cronbach’s *α* = 0.815.

## Results

A total of 44 knees (in 10 males and 34 females; mean age of 73.3 years) were evaluated with CT scans obtained both before and after surgery. A total of 20 knees were in the HM group and 24 were in the VM group. We found no significant differences in age, sex, preoperative diagnosis, K–L grade, preoperative FTA, or type of cement between the two groups. Mean preoperative HU values were 300 ± 132 HU in the HM group and 307 ± 142 HU in the VM group on the sclerotic side, and 80 ± 55 HU in the HM group and 73 ± 49 HU in the VM group on the nonsclerotic side; the differences between the two groups were not significant (Table 1).

**Table 1:** Demographic data, cement type, and preoperative Hounsfield unit values of bone penetration area

Characteristic	HM group (*n* = 20)	VM group (*n* = 24)	*p* value
Age (years)	72.3 ± 6.8	74.1 ± 7.2	0.21
Female, *n* (%)	14 (70%)	20 (83%)	0.33
Diagnosis			
Osteoarthritis	20	23	0.35
Osteonecrosis	0	1	
K–L grade 1/2/3/4	0/1/2/17	0/1/7/15	0.26
Pre-FTA (degrees)	183.6 ± 6.2	183.7 ± 7.5	0.95
Cement type (cobalt HV or cobalt G-HV)	17/3	15/9	0.10
Preoperative HU value			
Sclerotic side	300 ± 132	307 ± 142	0.89
Nonsclerotic side	80 ± 55	73 ± 49	0.70

*HU* Hounsfield unit, *FTA* femorotibial angle, *HM* hand mixing and hand packing, *VM* vacuum mixing and use of a cement gun, *K–L* Kellgren–Lawrence, *HV* bone cement without gentamicin, *G-HV* bone cement with gentamicin

The mean depths of penetration were 2.0 ± 0.7 mm in the HM group and 2.5 ± 0.7 mm in the VM group at the center of the sclerotic side; the difference was significant (*p* = 0.02). No significant differences were observed in the anterior and posterior areas of the sclerotic side or any areas of the nonsclerotic side. In the evaluation of the sagittal section, the mean penetration depths on the sclerotic side were 1.9 ± 0.5 and 2.2 ± 0.5 mm in the HM and VM groups, respectively (*p* = 0.054) (Table 2). Similarly, the mean maximum depths of penetration were 4.0 ± 0.9 and 5.0 ± 1.6 mm in the HM and VM groups at the center of the sclerotic side (*p* = 0.02), but they did not differ significantly in the anterior and posterior areas of the sclerotic side or any area of the nonsclerotic side. In the evaluation of the 3D CT image, the maximum penetration depths on the sclerotic side were 4.8 ± 0.4 and 5.9 ± 1.1 mm in the HM and VM groups (*p* = 0.008), respectively. On the nonsclerotic side, there were no significant difference were found in either sagittal section or 3D CT (Table 3). In the same cases, both the mean and maximum penetration depths were significantly smaller on the sclerotic side than on the nonsclerotic side.

**Table 2:** Mean depths of cement penetration (mm)

Side	Coronal plane	HM group	VM group	*p* value
Sclerotic	Anterior	2.1 ± 0.7	2.2 ± 0.5	0.46
	Central	2.0 ± 0.7	2.5 ± 0.7	0.02*
	Posterior	2.0 ± 0.7	2.2 ± 0.8	0.55
Nonsclerotic	Anterior	3.0 ± 0.9	2.9 ± 0.8	0.90
	Central	3.1 ± 1.0	3.2 ± 0.8	0.62
	Posterior	2.2 ± 0.7	2.1 ± 0.9	0.67

Side	Sagittal plane	HM group	VM group	*p* value
Sclerotic		1.9 ± 0.5	2.2 ± 0.5	0.05
Nonsclerotic		2.8 ± 0.6	2.8 ± 0.6	0.97

*HM* hand mixing and hand packing, *VM* vacuum mixing and use of a cement gun

**Table 3:** Maximum depths of cement penetration (mm)

Side	Coronal plane	HM group	VM group	*p* value
Sclerotic	Anterior	3.6 ± 0.9	3.9 ± 1.0	0.31
	Central	4.0 ± 0.9	5.0 ± 1.6	0.02*
	Posterior	3.6 ± 1.1	4.3 ± 1.5	0.10
Nonsclerotic	Anterior	4.6 ± 1.8	4.8 ± 1.5	0.75
	Central	4.7 ± 1.3	5.6 ± 1.7	0.06
	Posterior	3.8 ± 1.2	4.0 ± 1.8	0.67

Side	3D CT image	HM group	VM group	*p* value
Sclerotic		4.8 ± 0.4	5.9 ± 0.4	0.01*
Nonsclerotic		5.5 ± 1.4	5.8 ± 1.0	0.39

*HM* hand mixing and hand packing, *VM* vacuum mixing and use of a cement gun, *3D CT* three-dimensional computed tomography

**Fig. 5:**
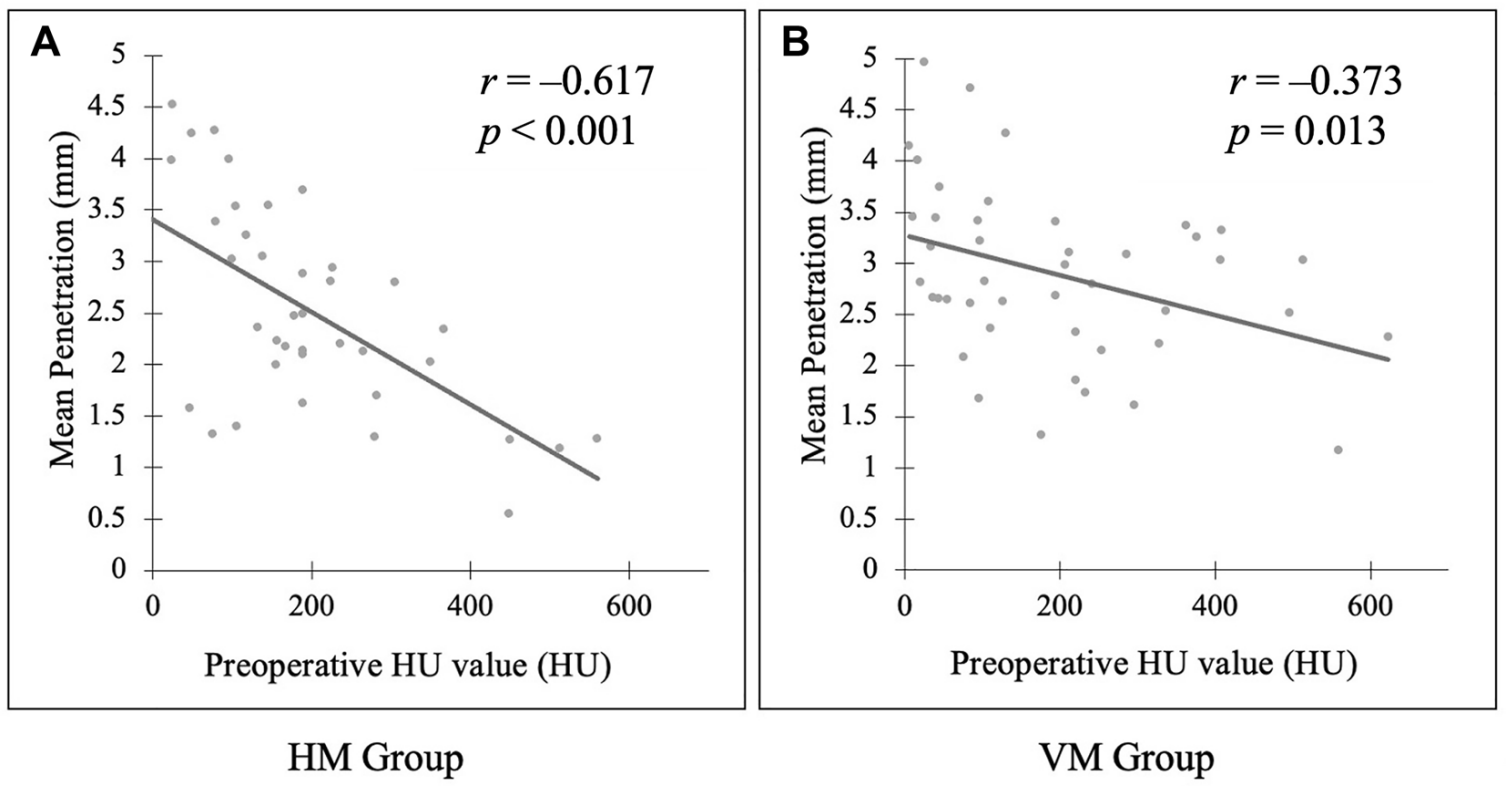
Correlation between preoperative Hounsfield unit (HU) value of bone penetration area and mean depth of penetration. **A** Correlation between bone penetration and preoperative HU values in the HM group. **B** Correlation between bone penetration and preoperative HU values in the VM group. In both groups, the correlation between preoperative HU values and mean penetration was negative. The negative correlation was larger in the HM group than in the VM group. *HM* hand mixing + hand packing, *HU* Hounsfield unit, *VM* vacuum mixing + cement gun

The correlation between preoperative HU values and mean penetration was negative in both groups: *r* = −0.617 (*p* < 0.01) in the HM group and *r* = −0.373 (*p* = 0.01) in the VM group (Fig. 5). We did not find evidence of early aseptic loosening or infection 2 years after TKA.

In the preliminary study using a bone model, the mean depths of penetration were 2.1 ± 0.4 mm in the cobalt HV group and 2.0 ± 0.3 mm in the cobalt G-HV group (*p* = 0.73), and the maximum depths of penetration were 4.8 ± 0.8 mm in the cobalt HV group and 4.8 ± 1.8 mm in the cobalt G-HV group (*p* = 0.50). No significant difference was found between the two groups.

## Discussion

We investigated the correlation between preoperative HU values and depths of cement penetration in TKA and we compared the depths of penetration under the tibial baseplate by technique. In this study, cement penetration was generally good in both groups; however, in the coronal section evaluation, the mean and maximum depths of penetration were larger in the center of the sclerotic side in the VM group. In the sagittal section evaluation, the mean penetration depth tended to be larger in the VM group than in the HM group. In the 3D CT evaluation, the maximum penetration depths were larger on the sclerotic side in the VM group. The negative correlation between preoperative HU values and depths of penetration was larger in the HM group. These results imply that it is difficult for cement to penetrate sclerotic bone, but the use of vacuum mixing and cement guns increased the depths of penetration.

Vanlommel et al. [16] reported that a cement penetration depth of 3–5 mm is optimal for implant stability and in preventing thermal injury to trabecular bone. Hampton et al. [17] reported that a penetration depth of less than 2 mm under the tibial component, shown on postoperative TKA radiographs, is a risk factor for aseptic loosening. Therefore, increasing the depth of penetration can improve the survival rate of TKA. The CT-based evaluation method of Verburg et al. [6] does not measure the vertical distance of cement penetration, whereas our method uses slices of coronal to evaluate the vertical distance. In addition, by using bone morphometric techniques, we measured not only the maximum depths of penetration but also the mean depths of penetration anteriorly, centrally, and posteriorly, which is a more reliable method of measurement. Furthermore, this study distinguishes itself from previous reports because, in addition to the evaluation of CT coronal sections, it also evaluated sagittal sections and 3D CT.

Various techniques have been reported to increase cement penetration, such as pulse lavage before cementation, drying the osteotomy surface, drilling anchor holes, using tourniquets, and using high-pressure carbon dioxide gas to remove blood and fat from the osteotomy surface [11, 18, 19]. The osteotomy surface must be treated before implant placement. Applying negative pressure with a tube to the stem insertion site [20] and using a cement gun to pressurize the osteotomy surface during cementation [21] can also increase cement penetration. The cement gun that we used in this study has a nozzle that can be easily press-fitted into the osteotomy surface, and we believe that this design enabled greater penetration.

Han et al. [22] reported the usefulness of finger packing pressure in TKA, and pressurizing even by manual method is important to increase cement penetration. On the other hand, one of the advantages of using a cement gun is that it can be applied directly to the implant and osteotomy surface without waiting for the cement to dry, whereas manual cement application requires waiting until the cement does not stick to the glove. Early application of cement reportedly increased the strength of the bone–cement interface [23], and in this study, the cement gun proved to be useful because the penetration of the sclerotic side was greater in the VM group. On the other hand, it has been reported that the use of a cement gun alone is not sufficient for fixing the tibial component [24]. The use of various techniques is important for increasing cement penetration.

Van de Groes et al. [25] showed that the bone–cement interface is weaker with cortical bone than with cancellous bone, which indicates that treatment for sclerotic bone is necessary. However, we have found no reports of the correlation between bone sclerosis and cement penetration or of the usefulness of cement guns for sclerotic bone. Because cement penetration decreases as bone sclerosis progresses, and because the penetration of sclerotic bone was greater in the VM group than in the HM group, the use of a cement gun may have been helpful. Although the use of a cement gun increases the short-term cost, it may reduce long-term cost because it increases cement penetration, thereby reducing the need for revision surgery. Moreover, compared with hand packing, a cement gun can apply cement to the implant without direct touch, which can help prevent infection.

## Limitations

This study included only a small number of cases. However, the analyzed power, 0.804, was based on a given *α* value, sample size, and an effect size of 0.770, which was determined from the mean and standard deviation of the depths of cement penetration in each group (calculated by the G*Power 3 power analysis program). This result means that the data are reliable. Second, long-term follow-up was not available, and long-term survival of implants is the optimal primary outcome. However, the purpose of this study was to investigate the difference in cement penetration by the cementing technique. Further investigation is needed to determine the long-term outcomes of the patients in this study.

## Conclusions

CT-based evaluation before and after TKA revealed that the cementing technique of vacuum mixing and using a cement gun enabled deeper cement penetration compared with the hand mixing and hand packing technique, even in sclerotic sites. We believe that compared with hand mixing and hand packing, this technique is less affected by the degree of bone sclerosis reflected by preoperative HU value.

## Data Availability

The data are not available for public access because of patient privacy concerns, but they are available from the corresponding author on reasonable request.

## Abbreviations

TKA: Total knee arthroplasty
CT: Computed tomography
HM: Hand mixing and hand packing
VM: Vacuum mixing and the use of a cement gun
K-L: Kellgren–Lawrence
FTA: Femorotibial angle
ICC: Intraclass correlation coefficient
HU: Hounsfield unit
